# Epigenetic Mechanisms Involved in Cisplatin-Induced Nephrotoxicity: An Update

**DOI:** 10.3390/ph14060491

**Published:** 2021-05-21

**Authors:** Pía Loren, Nicolás Saavedra, Kathleen Saavedra, Tomás Zambrano, Patricia Moriel, Luis A. Salazar

**Affiliations:** 1 Center of Molecular Biology and Pharmacogenetics, Scientific and Technological Bioresource Nucleus, Universidad de La Frontera, Temuco 4811230, Chile; pia.loren@ufrontera.cl (P.L.); nicolas.saavedra@ufrontera.cl (N.S.); kathleen.saavedra@ufrontera.cl (K.S.); 2 Department of Medical Technology, Faculty of Medicine, Universidad de Chile, Santiago 8380453, Chile; tomas.zambrano@uchile.cl; 3 Faculty of Pharmaceutical Sciences, University of Campinas, Campinas 13083970, SP, Brazil; patricia.moriel@fcf.unicamp.br

**Keywords:** epigenetics, non-coding RNA, DNA methylation, histone modification, nephrotoxicity

## Abstract

Cisplatin is an antineoplastic drug used for the treatment of many solid tumors. Among its various side effects, nephrotoxicity is the most detrimental. In recent years, epigenetic regulation has emerged as a modulatory mechanism of cisplatin-induced nephrotoxicity, involving non-coding RNAs, DNA methylation and histone modifications. These epigenetic marks alter different signaling pathways leading to damage and cell death. In this review, we describe how different epigenetic modifications alter different pathways leading to cell death by apoptosis, autophagy, necroptosis, among others. The study of epigenetic regulation is still under development, and much research remains to fully determine the epigenetic mechanisms underlying cell death, which will allow leading new strategies for the diagnosis and therapy of this disease.

## 1. Introduction

Globally, cancer is the second leading cause of death. In 2018, 18.1 million people worldwide had cancer, and 9.6 million died from this disease [1]. Cisplatin [*cis*-diamminedichloroplatinum (II), CDDP] is a well-known chemotherapeutic drug used for the treatment of numerous human cancer in solid organs, including head and neck [2], testis [3], small cells [4] and non-small cells [5] lung cancer, ovarian [6], cervical [7] and bladder [8]. Regarding the treatment of cancer cells, CDDP is potentially accompanied by some side effects such as ototoxicity, gastrotoxicity, neurotoxicity, myelosuppression, and allergic reactions [9]. Still, the main limiting side effect of CDDP use is nephrotoxicity [10]. Nephrotoxicity is defined as the rapid deterioration in kidney function due to the toxic effect of drugs, medications, and/or chemicals [11]. Cisplatin-induced-nephrotoxicity can occur in several ways, but the most severe and common is acute kidney injury, whose incidence after a single dose of CDDP fluctuates between 20–30% in patients who receive an initial dose of 50–100 mg/m^2^ [9]. Cisplatin is concentrated and reabsorbed by tubular renal cells (5 times more than in the blood), which triggers a rapid decrease in renal function [12], resulting in morphological changes and altered signaling pathways, such as vacuolation, mitochondrial dysfunction, cell cycle arrest and apoptosis [13,14]. The diagnosis of kidney damage is carried out using standard biomarkers, such as serum creatinine (sCr) and blood urea nitrogen (BUN). However, their sensitivity and specificity are very low, and values are altered when significant kidney damage already exists [15].

In recent years, several studies showed that epigenetic processes are involved in cisplatin-induced nephrotoxicity. Wu and Morris [16] defined epigenetic as the “study of the changes in gene expression, which occur in organisms with differentiated cells, and the mitotic inheritance of given patterns of gene expression”. These modifications regulate gene expression without changing the primary DNA sequence, triggering activation of transcription or gene silencing [17]. The main epigenetic mechanisms are represented by DNA methylation, histone modifications, and non-coding RNA. In general, epigenetic modifications are stable and heritable during cell divisions; however, they are potentially reversible and can be influenced by environmental factors. This review discusses recent findings in the epigenetics landscape of cisplatin-induced nephrotoxicity, focusing on the three mechanisms mentioned above.

## 2. Induction Mechanism Cisplatin-Induced Nephrotoxicity

Despite its therapeutic efficacy, the use of CDDP is limited due to the manifestation of side effects in normal tissues, mainly nephrotoxicity, whose incidence fluctuates between 20–30% in patients undergoing CDDP chemotherapy [9].

Cisplatin exerts its cytotoxic consequences by reacting with DNA, which ultimately culminates in irreversible apoptosis. Once CDDP goes through the cytosol, the low concentration of chloride present triggers the hydrolysis of the Pt-Cl bonds. One of the Cl ligands is replaced by H_2_O to form the first positively charged CDDP derivative [18]. The subsequent hydrolysis results in the replacement of the next Cl ligand, leading to the formation of the second CDDP derivative [18]. Cisplatin hydrolyzed complexes bind to negatively charged DNA bases, inducing DNA damage by forming different types of adducts, resulting in the arrest of the cell cycle. Cisplatin interacts primarily with the N7 sites of purine residues in DNA to form inter-strand DNA crosslinks and intra-strand crosslinks at 5′-GG-3′ sites of DNA bases. This results in defective DNA strands, causing the arrest of DNA synthesis and replication [19]. The main molecular sensor of DNA damage produced by CDPP is ATR (ataxia telangiectasia and Rad3-related), which is recruited to the site of damage and is co-localized with H2AX, forming nuclear foci. This is continued by the recruitment and activation of protein kinases, such as Chk1 and Chk2, which phosphorylate p53 [20], activating the signaling cascade that leads to cell death by apoptosis [21,22,23,24,25,26] or necrosis [27,28,29]. Although this way of DNA damage occurs in all cells, especially those that divide rapidly, such as tumor cells, this is considered the main mechanism mediating CDDP antitumor activity. CDDP cytotoxicity is not limited to cancer cells. In normal cells may lead to dangerous adverse effects such as ototoxicity, gastrotoxicity, myelosuppression, and allergic reactions. However, the major negative side effect of CDDP corresponds to nephrotoxicity [10]. After a single dose of CDDP (50–100 mg/m^2^), approximately 30% of patients develop this pathology [9]. The nephrotoxic effect is produced by CDDP accumulation in the kidney, mainly in the S3 segment of the proximal tubules [30]. One of the reasons for its accumulation may be determined by the high density of negatively charged mitochondria in the proximal tubular cells, which attracts positively charged CDDP hydrolyzed complexes [31]. Once CDDP enters the cell, it can induce various mechanisms resulting in cell damage. The intrinsic pathway is the main apoptotic pathway during CDDP-induced nephrotoxicity. Cisplatin decreases anti-apoptotic proteins such as Bcl-2, Bcl-XL, and Mcl-1 [32,33]. This triggers external membrane permeabilization of the mitochondria, releasing apoptotic factors, such as cytochrome *c* [34]. Once cytochrome *c* is released, recruitment and activation of caspase-9 occurs, therefore activating caspase-3, initiating the process of caspase-dependent apoptosis [35]. Cisplatin treatment also activates p53 [36,37,38,39], which phosphorylation results in the activation of several molecules, such as p53-up-regulated modulator of apoptosis (PUMA-α) and P53-induced protein with a death domain (PIDD) [21]. PIDD activates caspase-2, releasing apoptosis-inducing factor (AIF) from the mitochondria, resulting in caspase-independent activation [19]. On the other hand, PUMA-α translocates into mitochondria and interacts with BCL-XL, while BAX and BAK finally produce the release of cytochrome *c*, leading to caspase-dependent apoptosis [32,33]. Additionally, caspase-6 and -7 induction are produced during CDDP treatment [40]. Cisplatin also activates three major MAPKs: extracellular signal-regulated kinase (ERK), p38, and c-JUN N-terminal kinase (JNK) [35,41]. This induces oxidative stress, activating TNF-α and the consequent inflammatory response during CDDP-induced nephrotoxicity [42]. The augmented expression of ERK1/2 by CDDP treatment also activates NF-κβ and p53 signaling [43]. TNF-α increases tumor necrosis factors 1 and 2, TNFR1 and TNFR2, triggering the extrinsic apoptosis pathway through the activation of caspase-8, which will eventually lead to caspase-3 activation [9]. Cisplatin also regulates p21; its up-regulation protects from nephrotoxic damage [21]. Oxidative stress is a key factor contributing to nephrotoxicity since CDDP can react with glutathione, decreasing non-enzymatic (GSH and NADPH) and enzymatic (SOD, CAT, GPx, among others) antioxidant defense mechanisms [44]. Cisplatin also affects the mitochondrial respiratory complexes, leading to the inhibition of complexes I to IV of the respiratory chain and decreasing intracellular ATP levels [45]. In addition, CDDP also reduces the number of mitochondria in normal renal cells [43]. Finally, CDDP causes the activation of an adaptative program in the endoplasmic reticulum (ER) known as unfolded protein response (UPR), which inositol-requiring enzyme-1 (IRE1), double-stranded RNA-activated protein kinase-like ER kinase (PERK), and activating transcription factor-6 (ATF6) are dissociated from ER chaperone GRP78, activating signal transduction to inhibit protein translation [46]. Therefore, cisplatin-induced nephrotoxicity is a multifactorial and complex process involving multiple signaling pathways that in turn, may also be regulated by epigenetic mechanisms. 

## 3. DNA Methylation Role in Cisplatin-Induced Renal Dysfunction

**Figure 1:**
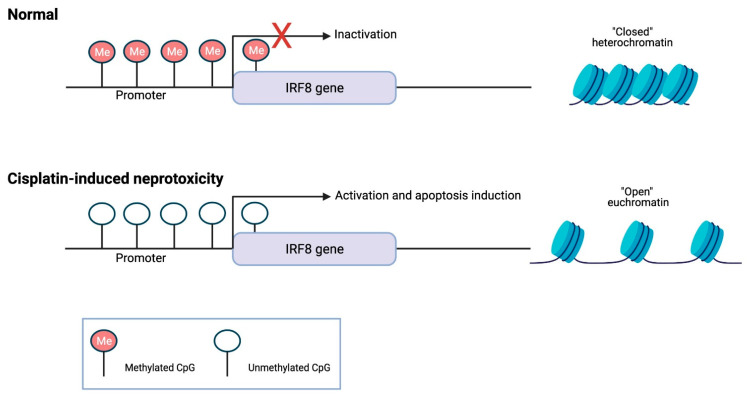
Demethylation of IRF8 gene promoter during cisplatin-induced nephrotoxicity, contributing to renal tubular cell apoptosis. Created with BioRender.com accessed on 21 May 2021.

DNA methylation is an epigenetic modification involving the covalent addition of a methyl group to carbon 5 of a cytosine at CpG dinucleotides through the action of DNA methyltransferase enzymes (DNMTs) [47], which include DNMT1, DNMT2, DNMT3A, DNMT3B, and DNMT3L, contributing to hypermethylated (high methylation levels) or hypomethylated (low methylation levels) states of genes or genomic regions containing CpG residues [48]. In mammals, 70–80% of CpG dinucleotides are methylated, whereas CpG islands in promoter regions of genes are demethylated, allowing transcription to proceed [49]. Therefore, the main function of DNA methylation is the regulation of transcription. Thus, hypomethylation in the promoter region correlates with gene activation by increased access for transcription factors, whereas hypermethylation in the promoter region results in loss of gene expression. Studies associating different DNA methylation patterns with CDDP-induced nephrotoxicity are scarce. Reports show that treatment of tubular cells with 5-aza-2′-deoxycytidine (5-AZA), a DNA methylation inhibitor, increases CDDP-induced apoptosis [50,51]. In addition, IRF8, a pro-apoptotic factor, is hypomethylated and induced after CDDP treatment, contributing to renal tubular cell apoptosis (Figure 1) [50]. In conjunction with 5-AZA, cisplatin decreases the expression of DNMT1, a maintenance DNMT, attenuating CDDP-induced nephrotoxicity [52]. It is recognized that OCT2, a transporter involved in organic cation transport, can transport CDDP to proximal tubules [53]. Based on this, studies show that methylation of the promoter region of OCT2 dramatically reduces the transcriptional activity of this transporter [54], which may contribute to increased toxicity.

## 4. Cisplatin Nephrotoxicity and Histone Modifications

The dynamic changes in chromatin structure, which allow its decondensation and remodeling, are processes necessary for gene transcription, DNA repair, and replication, carried out by post-translational modifications in the different histones [55]. Histones are the basic structural proteins of nucleosomes, which bind to double-helical DNA, forming the DNA-histone complex [47]. There are several types of histones, such as linker histones (H1 and H5) and core histones (H2A, H2B, H3, and H4), whose function is associated with DNA packaging [49], where CDDP can easily react with H1 at methionine and glutamate residues, forming tertiary complexes that prevent DNA repair and increase sensitivity to CDDP [56]. Histone modifications occur predominantly at the N-terminal end, changing chromatin structure, positively or negatively affecting gene expression [49]. There are at least 8 histone modifications, but the most studied include histone acetylation and methylation, mostly in lysine and arginine residues. 

**Figure 2:**
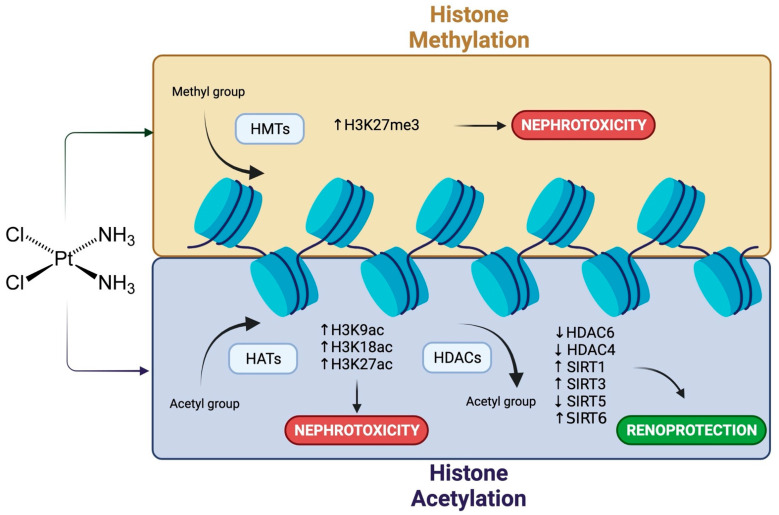
Proposed role of histone modifications in the regulation involved in cisplatin-induced nephrotoxicity. Created with BioRender.com accessed on 21 May 2021.

Histone methylation involves the addition of a methyl group to a basic amino acid (lysine, arginine, and rarely in histidine) of core histones by a group of enzymes called histone methyltransferases (HMTs) [49]. Generally, methylation at H3 lysine 4 (H3K4), H3K36, and H3K79 activate gene transcription, whereas methylation at H3K9, H3K27, and H4K20 is associated with transcriptional repression [47]. CDDP treatment increases H3K27 trimethylation, leading to increased caspase 3 and decreased cell viability (Figure 2) [35]. Additionally, inhibition of enhancer of zeste homolog 2 (EZH2) -a histone methyltransferase- with 3-deazaneplanocin A (DZNep) in rat proximal tubular cells aggravates apoptosis by decreasing mTOR complexes [57].

On the other hand, histone acetylation involves adding an acetyl group to the lysine residue of core histones by a group of enzymes called histone acetyltransferases (HATs) [47]. This promotes open chromatin because a negative charge is added to the positively charged lysines, reducing the strong DNA-histone interaction, and thus, gene expression is activated [58]. One member of HATs is p300. This HAT can directly bind to transcription factors, such as p53 or NF-κB, and regulate their activities by acetylation [59,60]. Cisplatin induces p300 activation, acetylating lysines 18, 27, and 9 on H3, triggering functional and histological injury, increasing oxidative stress, inflammation, and apoptosis (Figure 2) [61]. One strategy to decrease CDDP-induced damage has been the inhibition of p300 by garcinol, attenuating the oxidative stress, inflammation, and apoptosis, by reducing the acetylation of the p65 subunit of NF-κB [61].

Acetylation is a reversible process catalyzed by enzymes called histone deacetylases (HDACs) [49]. HDACs are subdivided into four types: class I (HDAC1, 2, 3, and 8), class IIa (HDAC4, 5, 7, and 9), class IIb (HDAC6 and 10), class III (SIRT1-7), and class IV (HDAC11) [58]. One of the strategies to decrease CDDP-induced nephrotoxicity has been the use of HDACs class II inhibitors, as their overexpression is associated with increased apoptosis [62,63]. Studies have reported that the level of acetylated histone H3 is modified after CDDP treatment, but CDDP does not alter the abundance of histone H4 [64]. However, controversial results have been reported concerning acetylation of histone H3 with CDDP treatment. On the one hand, CDDP reduces levels of histone H3 acetylation in the injured kidney tissue [46,65]; however, another study demonstrated an increase in histone H3 acetylation after CDDP treatment in human renal cortical epithelial (HRCE) cells [66]. Although HDACs inhibitors do not prevent the DNA adducts formation and the initial damage response, they can interfere with the subsequent signaling [67]. Inhibition of HDAC6 by N-hydroxy-4-(2-methoxy-5-(methyl(2-methylquinazolin-4-yl)amino)phenoxy)butanamide (23BB) reduces apoptosis and ER stress in the kidney of cisplatin-injured mice [46]. Additionally, HDAC6 inhibition by trichostatin A (TSA) reduces the renal pathological damage caused by CDDP treatment [65]. Finally, reduction of HDAC4 and HDAC5 with LMK-235 blocks the caspase-3 cleavage in HRCE cells [66]. The use of TSA, valproate (VPA) or suberoylanilide hydroxamic acid (SAHA) can confer kidney protection during CDDP treatment by H3K27 acetylation and IL-9 down-regulation [62], increase of BMP-7 [63], AMWAP [68,69] and PSTPIP2 [70]; increased CREB phosphorylation [71], reduction of caspase-3 activation [71] or by autophagy stimulation [72]. Even more, it has been demonstrated that SAHA inhibits and TSA delays phosphorylation, acetylation, and activation of p53 in rat proximal tubular cells [67]. On the other hand, overexpression of HDAC class III decreases CDDP-induced damage. Pharmacological overexpression of SIRT1 ameliorates the increased acetylation of p65 of NF-κB [73] and p53 [74] during cisplatin treatment, which decreases apoptosis, oxidative stress, and inflammation [64,75]. However, SIRT2 up-regulation increases phosphorylation of p38 and JNK, inducing apoptosis and renal damage in renal tissue [76]. In the same way, SIRT6 overexpression attenuates cisplatin-induced damage by repressing the expression of ERK1/2, thus inhibiting NF-κB and p53 signaling [43]. Finally, SIRT3 activation protects kidney tubular cells against oxidative stress by PARP1 inhibition [77], and its deficiency augments CDDP nephrotoxicity by increasing apoptosis and inflammatory response (Figure 2) [78]. However, silencing SIRT5 expression significantly improves kidney function and decreases tissue damage in mice submitted to CDDP damage [79].

## 5. Involvement of Non-Coding RNAs in Cisplatin Nephrotoxicity

Less than 2% of the human genome is transcribed into RNA to be translated into proteins. The remaining 98% is transcribed into a class of RNA that is not translated into proteins but plays a critical role in epigenetics. These molecules are called non-coding RNAs (ncRNAs) [47]. They regulate gene expression under physiological and pathological conditions, acting at various stages in protein synthesis at both transcriptional and post-transcriptional levels [80]. Non-coding RNAs are further divided into two main groups, small ncRNAs (shorter than 200 bp) and long ncRNA (lncRNAs; longer than 200 bp). 

Out of small ncRNAs, microRNAs (miRs) have been by far the most extensively studied. They are a class of short-chain, linear, approximately 21–25 nucleotides long that negatively regulate gene targets at the post-transcriptional level by perfect complementarity of their “seed” region to 3′-UTR of its target mRNA, inducing their degradation. If there is a mismatch or imperfect complementarity, it results in translational repression [49]. The latest release of the miRbase database (v22) contains 2654 human mature miRs sequences [81], ratifying their importance on gene expression regulation. 

**Figure 3:**
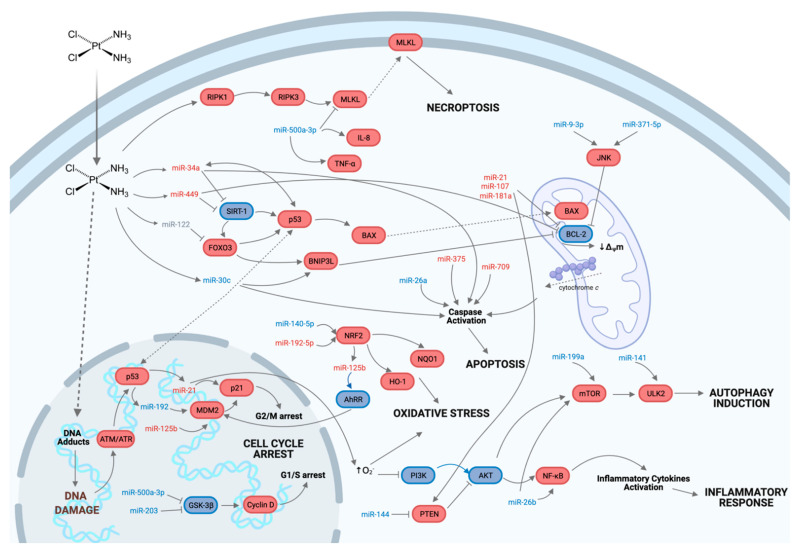
Proposed model of miRs function in the activation of different signaling pathways during cisplatin-induced nephrotoxicity in rodent and human tubular cells. Block arrows indicate inhibitory effect; single arrows indicate stimulatory effect. The blue color indicates gene expression down-regulation, while the red color indicates gene expression up-regulation. Created with BioRender.com accessed on 21 May 2021.

Evidence shows that miRs mediate CDDP-induced nephrotoxicity in renal tubular cells through modulation of several molecules involved in a wide array of human (Table 1) and rodents (Table 2) signaling pathways, activating mechanisms such as DNA damage response, oxidative stress, autophagy, necroptosis, cell cycle arrest, apoptosis and inflammatory response (Figure 3). Cisplatin induces apoptosis in renal tubular cells [21,22,23,24,25,26], resulting in p53 activation. However, the epigenetic mechanism regulating cellular death is not well defined. Bhatt et al. demonstrated that induction of miR-34a in vitro during CDDP treatment in BLUMPT cells and in vivo during CDDP-induced nephrotoxicity in C57BL/6 mice were generated by p53. Still, this induction was abrogated in p53-deficient mice [21]. However, other studies showed that CDDP treatment down-regulates miR-122 expression, causing FOXO3 acetylation and activation [36]; simultaneously, miR-34a expression is induced, causing SIRT1 repression [36,37] together with triggering p53 activation [36]. FOXO3 activation also increases BNIP3L expression, a process that is mediated by miR-30c repression [22]. SIRT1 repression is also preceded by miR-449 up-regulation [32]. Upon CDDP-induced damage and consequent p53 activation, BAX is translocated into the mitochondria causing BCL-2 down-regulation, a process mediated by miR-181a [33], miR-449 [32], miR-21-5p and miR-200c [82]; and miR-107 [83]. CDDP treatment has also shown JNK induction [84], which promotes the apoptotic process. The decrease in mitochondrial membrane potential as a consequence of the increased BAX/BCL-2 ratio leads to the release of cytochrome *c* into the cytosol, binding to other molecules to form the apoptosome and initiating the caspase activation cascade, which is modulated by miRs such as miR-26a [25], -30c [22], -34a [21], -375 [85] and -709 [23].

Although autophagy is generally activated under conditions of nutrient deprivation, it has also been implicated in physiological (development, differentiation) and pathological (neurodegenerative diseases, stress, infection, cancer) processes [86]. Based on this, it has been proven that CDDP also modulates the autophagy process in renal tubular cells [87,88], although the epigenetic mechanisms underlying its occurrence have been poorly studied. However, it is known that CDDP modulates MAPK/ERK1/2 signaling pathway through miR-146b up-regulation [89]. Enhanced mir-146b levels directly impact the expression of mTOR, an autophagy initiation regulatory kinase, which is in turn mediated by miR-199a up-regulation [38] and miR-26b down-regulation [90]. This allows the interaction of Atg13, FIPP200, and ULK1/2 [91], the last one being regulated by miR-141 repression [92]. The Atg13/FIPP200/ULK composite interacts with the PI3K class III complex, composed of several molecules, including Beclin-1, which recruit several ATG proteins, allowing the phagophore formation [91], and thus, starting the autophagy process. 

Cisplatin also modulates cellular oxidative stress by regulating NRF2 [93], a transcription factor that regulates the expression of genes encoding antioxidant, anti-inflammatory, and detoxifying proteins [94]. Under normal conditions, NRF2 is located in the cytoplasm, negatively controlled by KEAP1; whereas, under oxidative stress conditions, KEAP1 releases NRF2, which can be translocated to the nucleus and, in this way, binds to antioxidant response elements (ARE), resulting in the transcription of antioxidant genes [95]. Some miRs have been shown to modulate KEAP1/NRF2 interaction, such as miR-192-5p [24] and miR-140-5p [93]. In addition to this, CDDP also alters the redox state, by decreasing the antioxidant activity of GSH, GSH-Px, and SOD [37].

Necroptosis is a programmed form of necrotic cell death considered passive cell death through regulated cell signaling pathways [96]. RIPK1, RIPK3, and MLKL are the main molecules involved in the necroptosis process. Necroptosis is triggered when TNFR1 is activated by TNF-α, resulting in complex I formation. Subsequently, RIPK1, TRADD, and FADD are recruited. Once RIPK1 is activated, its interaction with RIPK3 occurs, forming complex IIb/necrosome, which mediates the phosphorylation of MLKL, whose translocation to the plasma membrane promotes necroptosis by disrupting plasma membrane integrity [97]. Some studies in renal tubular cells have demonstrated the involvement of miR-500a-3p in RIPK1, RIPK3, and MLKL regulation when these cells are treated with CDDP [98,99]. On the other hand, RIPK1 ubiquitination results in NF-κB activation, mediating the survival and production of multiple inflammatory cytokines, which are also regulated by several miRs when these cells are exposed to CDDP. Thus, IL-6 is mediated by miR-494 [15] and miR-34a [37]; IL-8 by miR-500a-3p [99]; and finally, IL-1β by miR-155 [100] and miR-34a [37].

The functional and pathological roles of lncRNAs also have been implicated in CDDP-induced nephrotoxicity. The lncRNA XLOC_032768 can attenuate CDDP-induced apoptosis through TNF-α up-regulation [101]. Similarly, PRNCR1 lncRNA also attenuates CDDP-induced damage in HK-2 cells through up-regulation of EZH1 expression [102]. However, some lncRNAs have been implicated in nephrotoxic damage. Thus, during CDDP-induced damage, lncRNA LRNA9884 increases NF-κB-mediated inflammatory cytokines, especially MIF [103]. Likewise, lncRNA GAS5 can increase CDDP-induced apoptosis of HK-2 cells by modulating miR-205-5p [104].

Circular RNAs (circRNAs) are single-stranded, covalently locked transcripts produced from a precursor mRNA [105]. These circRNAs can act as sponges for miRs, resulting in increased expression [49]. In this way, circ-0114427 attracts miR-494 and interacts with it, increasing ATF3 expression and reducing inflammatory cytokines, thus exerting a nephroprotective role against CDDP damage [15].

## 6. Potential Challenges and Future Directions

Epigenetics is an active research field concerning cisplatin-induced nephrotoxicity. So far, studies show that the mechanisms involved play a significant role in the pathogenesis of this condition. However, little is known about the interaction between different epigenetic marks. Nonetheless, crosstalk between epigenetic modifications does exist in this pathology. For example, during CDDP-induced nephrotoxicity, lncRNA LRNA9884 increases NF-κB expression [103]. On the other hand, miR-34 [21,36], miR-192-5p [24] and, miR-449 [32] activate p53. Both NF-κB and p53 up-regulate miR-375 [85], inducing apoptosis in renal proximal tubular cells. However, inhibiting p300 attenuates this adverse effect due to reduced acetylation of NF-κB [61]. In the same way, overexpression of SIRT1 and SIRT6 inhibits p53 signaling, reducing cisplatin-induced damage [43,74]. Clearly, there are many gaps regarding epigenetic modulation in the pathogenesis of this condition, especially in the cross-talking for these mechanisms. Understanding the way that DNA methylation, histone modifications, and ncRNAs underlie this disease will provide us with the opportunity to discover new biomarkers for early diagnosis, as well as the establishment of novel therapies or treatments aiming to decrease the incidence of cisplatin-induced nephrotoxicity.

## 7. Conclusions

Nephrotoxicity is the most lethal side effect of cisplatin treatment. During the last few years, rapid progress has been made in understanding the contribution of epigenetic mechanisms underlying nephrotoxicity to be used as potential biomarkers for early diagnosis and/or to employ different therapeutic strategies. These studies demonstrate the complexity of the interactions of epigenetic modifications, which have provided a better understanding of the epigenetic regulation of CDDP-induced nephrotoxicity. Continued research in this area will provide new therapeutic targets and early, sensitive, and specific diagnostic biomarkers to detect CDDP-induced nephrotoxicity.

## Figures and Tables

**Table 1:** In vitro and in vivo evidence of microRNAs involved in cisplatin-induced nephrotoxicity in humans.

miRNA	Assay	Model/Cell Line	Source	CDDP Dose	Time	Effect	Exp.	Induced by	Gene Target	Ref.
miR-9-3p	in vitro	HK-2 cell line	PTECs	2, 10 or 50 µM	24 h	Toxic	Down	-	CASK	[84]
miR-18a-5p	in vitro	HPTEC cell line	PTECs	40 µM	6 and 24 h	Toxic	Down	-	-	[106]
miR-21	in vivo	Patients with malignant mesothelioma	Urine and kidney	CDDP chemotherapy	4–144 h	Toxic	Up	-	P21, BCL2	[82]
	in vitro	HPTEC cell line	PTECs	85 µM	24 h		Down	-	P21, BCL2	[82]
	in vitro	ciPTEC-OAT1	Cell culture media	5 to 30 µM	48 h	Toxic	Down	-	-	[39]
miR-26a	in vitro	HK-2 cell line	PTECs	4 µmol/L	24 h	Toxic	Down	-	TRPC6, DRP1	[25]
miR-29a	in vitro	ciPTEC-OAT1	Cell culture media	5–30 µM	48 h	Toxic	Up	-	-	[39]
miR-30c	in vitro	HK-2 cell line	PTECs	10 µM	24–72 h	Toxic	Down	-	BNIP3L, HSPA5	[22]
miR-31-5p	in vitro	HK-2 cell line	PTECs	40 µM	0–48 h	Toxic	Up	-	NUMB	[107]
miR-34a	in vitro	HK-2 cell line	PTECs	9 µg/mL	24 h	Toxic	Up	-	SIRT1	[37]
	in vitro	ciPTEC-OAT1	Cell culture media	5–30 µM	48 h	Toxic	Up	-	-	[39]
miR-107	in vitro	HK-2 cell line	PTECs	7.5 mM	6 h	Toxic	Up	-	RPS19	[83]
miR-125b	in vitro	HepG2, HEK-293 cell line	PTECs	30 µM	24 h	Protective	Up	NRF2	AhRR, MDM2	[108]
miR-140-5p	in vitro	HK-2 and 293T cell lines	PTECs	1.5–12 µM	24 h	Toxic	Up	-	NRF2, MnSOD, LDH	[93]
miR-146b-5p	in vitro	HPTEC cell line	PTECs	40 µM	6 and 24 h	Toxic	Down	-	-	[106]
miR-181a	in vitro	HK-2 cell line	PTECs	50 µmol/L	24 h	Toxic	Up	-	BCL2, BAX	[33]
miR-182	in vitro	HK-2 and 293T cell line	PTECs	20 µmol/L	24 h	Toxic	Down	-	FOXO1	[15]
miR-192	in vitro	ciPTEC-OAT1	Cell culture media	5–30 µM	48 h	Toxic	Up	-	-	[39]
miR-194-5p	in vitro	HK-2 cell line	PTECs	20 µM	24 h	Toxic	Up	-	-	[99]
miR-199a-3p	in vitro	HK-2 cell line	PTECs	100 µM	24 h	Toxic	Up	p53	mTOR	[38]
miR-200c	in vivo	Patients with malignant mesothelioma	Urine and kidney	CDDP chemotherapy	4–144 h	Toxic	Up	-	P21, BCL2	[82]
	in vitro	HPTEC cell line	PTECs	85 µM	24 h		Down	-	P21, BCL2	[82]
miR-203	in vitro	HK-2 and 293T cell line	PTECs	20 µmol/L	24 h	Toxic	Down	-	GSK-3β	[15]
miR-205	in vitro	HK-2 and HEK-293 cells	PTECs	100 µg/mL	6 h	Toxic	Down	-	CMTM4	[109]
miR-371b-5p	in vitro	HK-2 cell line	PTECs	2, 10 or 50 µM	24 h	Toxic	Down	-	CDK6	[84]
miR-423	in vivo	Patients with malignant mesothelioma	Urine and kidney	CDDP chemotherapy	4–144 h	Toxic	Up	-	P21, BCL2	[82]
	in vitro	HPTEC cell line	PTECs	85 µM	24 h		Down	-	P21, BCL2	[82]
miR-494	in vitro	HK-2 and 293T cell line	PTECs	20 µmol/L	24 h	Toxic	Down	-	ATF3	[15]
miR-500a-3p	in vitro	HK-2 cell line	PTECs	20 µM	24 h	Toxic	Down	-	MLKL, RIPK1, RIPK3	[99]
	in vitro	HK-2 cell line	PTECs	15 µM	6–48 h	Toxic	Down	-	MLKL, RIPK3	[98]
miR-577	in vitro	HK-2 cell line	PTECs	20 µM	24 h	Toxic	Up	-	-	[99]
miR-3168	in vivo	Patients with primary squamous cell carcinoma of the head and neck	Blood	CDDP chemotherapy	-	Toxic	Up	-	PRKAB2, TOP2A	[110]
miR-4718	in vivo	Patients with primary squamous cell carcinoma of the head and neck	Blood	CDDP chemotherapy	-	Toxic	Down	-	PRKAB2, ERCC1	[110]
miR-6125	in vivo	Patients with primary squamous cell carcinoma of the head and neck	Blood	CDDP chemotherapy	-	Toxic	Up	-	ERCC1	[110]

PTECs: Proximal Tubular Epithelial Cells; OAT1: Organic Anion Transporter 1.

**Table 2:** In vitro and in vivo evidence of microRNAs involved in cisplatin-induced nephrotoxicity in rodents.

miRNA	Specie	Assay	Model/Cell Line	Source	CDDP Dose	Time	Effect	Exp.	Induced by	Gene Target	Ref.
miR-9-3p	Rat	in vitro	NRK52E cell line	PTECs	2, 10 or 50 µM	24 h	Toxic	Down	-	CASK, CDK6, JNK	[84]
miR-15	Rat	in vivo	Male Han Wistar rat	Blood, urine and kidney	1 or 3 mg/kg	0–26 d	Toxic	Up	-	-	[111]
miR-16	Rat	in vivo	Male Han Wistar rat	Blood, urine and kidney	1 or 3 mg/kg	0–26 d	Toxic	Up	-	BTG2, PHLD3A	[111]
miR-20a	Rat	in vivo	Male Han Wistar rat	Blood, urine and kidney	1 or 3 mg/kg	0–26 d	Toxic	Up	-	p21	[111]
miR-21	Mouse	in vivo	Adult C57BL/6 mice	Urine, blood and kidney	15 mg/kg	4 d	Toxic	Up	-	-	[92]
		in vitro	MM55.K tubular cells	PTECs	8 µg/mL	48 h					
miR-22	Rat	in vivo	Male Sprague-Dawley rats	Urine	2 or 5 mg/kg	72 h	Toxic	Up	-	-	[112]
miR-26a	Mouse	in vivo	Male C57BL/6 mice	Kidney	20 mg/kg	3 d	Toxic	Down	-	TRPC6, DRP1	[25]
miR-26b	Rat	in vivo	Male Sprague-Dawley rats	Urine	2 or 5 mg/kg	72 h	Toxic	Up	-	-	[112]
			Male Wistar rats	Blood and kidney	5 mg/kg	3 d	Toxic	Down	-	TGFβR-1, TAK1, mTOR, LC3-II	[90]
miR-30c	Rat	in vivo	Wistar rat	Kidney	10 mg/kg	1, 3 or 7 d	Toxic	Down	-	BNIP3L, HSPA5	[22]
		in vitro	NRK-52E cell line	PTECs	10 µM	24–72 h					
miR-31-5p	Rat	in vivo	Female Sprague-Dawley rats	Blood and kidney	20 mg/kg	14 d	Toxic	Up	-	NUMB	[107]
miR-34a	Mouse	in vitro	BUMPT-306 cell line	PTECs	40 µmol/L	0–20 h	Toxic	Up	-	p53	[21]
	Mouse	in vivo	Wild-type and p53-deficientC57BL/6 mice	Blood and kidney	30 mg/kg	0–3 d					
	Mouse	in vivo	Male C57BL/6 mice	Kidney	15 mg/kg	3 d	Toxic	Up	-	SIRT1, p53, PHLDA3	[36]
	Rat	in vitro	NRK-52E cell line	PTECs	9 µg/mL	24 h	Toxic	Up	-	SIRT1	[37]
	RatMouse	in vivo	Male Wistar ratsMale C57BL-6J mice	Blood and kidney	7 mg/kg25 mg/kg	12 d			-	-	[37]
	Rat	in vivo	Male Sprague-Dawley rats	Kidney and plasma	2 or 5 mg/kg	72 h	Toxic	Up	-	-	[112]
	Rat	in vivo	Male Wistar rats	Blood and kidney	5 mg/kg	3 d	Toxic	Down	-	TGFβR-1, TAK1, mTOR, LC3-II	[90]
miR-34c	Rat	in vivo	Male Sprague-Dawley rats	Kidney and plasma	2 or 5 mg/kg	72 h	Toxic	Up	-	-	[112]
miR-34c-5p	Rat	in vivo	Male Hannover Wistar rats	Kidney and urine	2.5 mg/kg	0–27 d	Toxic	Down	-	-	[113]
miR-92a	Mouse	in vivo	Adult C57BL/6 mice	Urine, blood and kidney	15 mg/kg	4 d	Toxic	Down	-	-	[92]
		in vitro	MM55.K tubular cells	PTECs	8 µg/mL	48 h			-		
miR-92b	Rat	in vivo	Male Sprague-Dawley rats	Plasma	2 or 5 mg/kg	72 h	Toxic	Up	-	-	[112]
miR-93-5p	Rat	in vivo	Male Sprague-Dawley rats	Kidney and urine	1, 3 or 6 mg/kg	1–7 d	Toxic	Up	-	-	[114]
miR-107	Rat	in vivo	Male Sprague-Dawley rats	Kidney and blood	6 mg/kg	3 d	Toxic	Up	-	RPS19	[83]
miR-122	Mouse	in vivo	Male C57BL/6 mice	Kidney	15 mg/kg	3 d	Toxic	Down	-	FOXO3, p53, PHLDA3	[36]
miR-122-5p	Rat	in vivo	Male Sprague-Dawley rats	Blood and kidney	6 mg/kg	1–5 d	Toxic	Down	-	-	[115]
miR-125b	Mouse	in vivo	Male C57BL/6 mice and Nrf2 KO	Blood and kidney	15 mg/kg	3 d	Protective	Up	NRF2	AhRR, MDM2	[108]
miR-128	Rat	in vivo	Male Sprague-Dawley rats	Plasma	2 or 5 mg/kg	72 h	Toxic	Up	-	-	[112]
miR-130b	Rat	in vivo	Male Sprague-Dawley rats	Plasma	2 or 5 mg/kg	72 h	Toxic	Up	-	-	[112]
miR-134	Rat	in vivo	Male Sprague-Dawley rats	Plasma	2 or 5 mg/kg	72 h	Toxic	Up	-	-	[112]
miR-138	Mouse	in vivo	Female Diversity Outbred mice	Urine and kidney	5 mg/kg	3 d	Toxic	Up	-	-	[26]
miR-140	Rat	in vivo	Male Sprague-Dawley rats	Urine	2 or 5 mg/kg	72 h	Toxic	Up	-	-	[112]
miR-140-5p	Mouse	in vivo	Adult male mice	Blood and kidney	20 mg/kg	1–14 d	Toxic	Up	-	NRF2, MnSOD, LDH	[93]
		in vivo	Male C57BL/6J mice	Blood and kidney	20 mg/kg	19 d	Toxic	Down	-	NRF2, p53	[24]
miR-141	Mouse	in vivo	Adult C57BL/6 mice	Urine, blood and kidney	15 mg/kg	4 d	Toxic	Down	-	ULK2	[92]
		in vitro	MM55.K tubular cells	PTECs	8 µg/mL	48 h			-		
miR-143-3p	Rat	in vivo	Male Sprague-Dawley rats	Blood and kidney	6 mg/kg	1–5 d	Toxic	Down	-	-	[115]
miR-144	Mouse	in vivo	Male C57BL/6 mice	Blood and kidney	20 mg/kg	6 h	Toxic	Down	-	PTEN, ATK, GSk3β	[116]
	Rat	in vivo	Male Sprague-Dawley rats	Kidney	2 or 5 mg/kg	72 h	Toxic	Down	-	-	[112]
miR-146a	Mouse	in vivo	Male Balb/C mice	Blood and kidney	10 mg/kg	24 h	Toxic	Up	-	-	[117]
miR-146b	Rat	in vivo	Sprague-Dawley rats	Serum and kidney	6 mg/kg	5 d	Toxic	Up	-	ERBB4	[89]
		in vitro	NRK-52E cell line	PTECs	7.5 µM	48 h			-		
		in vivo	Male Sprague-Dawley rats	Kidney	2 or 5 mg/kg	72 h	Toxic	Up	-	-	[112]
miR-151	Rat	in vivo	Male Sprague-Dawley rats	Plasma	2 or 5 mg/kg	72 h	Toxic	Up	-	-	[112]
miR-155	Mouse	in vivo	miR-155^−/−^ and C57BL/6 mice	Blood and kidney	20 mg/kg	72 h	Toxic	Down	-	c-FOS, TNF-α, IL-1β	[100]
miR-181a	Mouse	in vivo	Male C57BL/6 mice	Blood and kidney	20 mg/kg	3 d	Toxic	Up	-	PTEN	[118]
miR-181b	Rat	in vivo	Male Sprague-Dawley rats	Plasma	2 or 5 mg/kg	72 h	Toxic	Up	-	-	[112]
miR-182	Mouse	in vivo	Male C57BL/6 mice	Blood and kidney	30 mg/kg	72 h	Toxic	Down	-	FOXO1	[15]
miR-191a	Rat	in vivo	Male Sprague-Dawley rats	Plasma	2 or 5 mg/kg	72 h	Toxic	Up	-	-	[112]
miR-191a-5p	Rat	in vivo	Male Sprague-Dawley rats	Kidney and urine	1, 3 or 6 mg/kg	1–7 d	Toxic	Up	-	-	[114]
miR-192	Rat	in vivo	Male Han Wistar rat	Blood, urine and kidney	1 or 3 mg/kg	0–26 d	Toxic	Up	-	-	[111]
miR-192-5p	Rat	in vivo	Male Sprague-Dawley rats	Kidney and urine	1, 3 or 6 mg/kg	1–7 d	Toxic	Up	-	-	[114]
	Mouse	in vivo	Male C57BL/6J mice	Kidney and blood	20 mg/kg	19 d	Toxic	Down	-	NRF2, p53	[24]
miR-193	Rat	in vivo	Male Han Wistar rat	Blood, urine and kidney	1 or 3 mg/kg	0–26 d	Toxic	Up	-	-	[111]
miR-199a-3p	Mouse	in vivo	Male C57/BL mice	Kidney	20 mg/kg	3 d	Toxic	Up	p53	mTOR	[38]
miR-203	Mouse	in vivo	Male C57BL/6 mice	Blood and kidney	30 mg/kg	72 h	Toxic	Down	-	GSK-3β	[15]
miR-208a	Mouse	in vivo	Adult C57BL/6 mice	Urine, blood and kidney	15 mg/kg	4 d	Toxic	Down	-	-	[92]
		in vitro	MM55.K tubular cells	PTECs	8 µg/mL	48 h			-		
miR-210	Rat	in vivo	Male Han Wistar rat	Blood, urine and kidney	1 or 3 mg/kg	0–26 d	Toxic	Up	-	-	[111]
miR-218	Mouse	in vivo	Female Diversity Outbred mice	Urine and kidney	5 mg/kg	3 d	Toxic	Up	-	-	[26]
miR-292-3p	Mouse	in vivo	Adult C57BL/6 mice	Urine, blood and kidney	15 mg/kg	4 d	Toxic	Down	-	-	[92]
		in vitro	MM55.K tubular cells	PTECs	8 µg/mL	48 h			-		
miR-293^*^	Mouse	in vivo	Adult C57BL/6 mice	Urine, blood and kidney	15 mg/kg	4 d	Toxic	Up	-	-	[92]
		in vitro	MM55.K tubular cells	PTECs	8 µg/mL	48 h			-		
miR-371b-5p	Rat	in vitro	NRK52E cell line	PTECs	2, 10 or 50 µM	24 h	Toxic	Down	-	CASK, CDK6, JNK	[84]
miR-375	Mouse	in vivo	Wild-type and PT-Dicer^−/−^ mice	Kidney	30 mg/kg	3 d	Toxic	Up	p53, NF-κB	HNF-1β	[85]
	Rat	in vitro	RPTC cell line	PTECs	20 µM	16 h					
miR-377	Mouse	in vivo	Adult C57BL/6 mice	Urine, blood and kidney	15 mg/kg	4 d	Toxic	Up	-	CUL1	[92]
		in vitro	MM55.K tubular cells	PTECs	8 µg/mL	48 h			-		
miR-378a	Rat	in vivo	Male Sprague-Dawley rats	Urine	2 or 5 mg/kg	72 h	Toxic	Up	-	-	[112]
miR-431	Rat	in vivo	Male Sprague-Dawley rats	Plasma	2 or 5 mg/kg	72 h	Toxic	Up	-	-	[112]
miR-449	Rat	in vitro	NRK-52E cell line	PTECs	20 µg/mL	24 h	Toxic	Up	-	SIRT1, P53, BAX	[32]
miR-451	Rat	in vivo	Male Sprague-Dawley rats	Kidney	2 or 5 mg/kg	72 h	Toxic	Down	-	-	[112]
miR-463	Mouse	in vivo	Adult C57BL/6 mice	Urine, blood and kidney	15 mg/kg	4 d	Toxic	Up	-	-	[92]
		in vitro	MM55.K tubular cells	PTECs	8 µg/mL	48 h			-		
miR-484	Rat	in vivo	Male Sprague-Dawley rats	Plasma	2 or 5 mg/kg	72 h	Toxic	Up	-	-	[112]
miR-494	Mouse	in vivo	Male C57BL/6 mice	Blood and kidney	30 mg/kg	72 h	Toxic	Down	-	ATF3, IL6	[15]
miR-673-5p	Mouse	in vivo	Adult C57BL/6 mice	Urine, blood and kidney	15 mg/kg	4 d	Toxic	Down	-	-	[92]
		in vitro	MM55.K tubular cells	PTECs	8 µg/mL	48 h			-		
miR-675-3p	Mouse	in vivo	Adult C57BL/6 mice	Urine, blood and kidney	15 mg/kg	4 d	Toxic	Up	-	-	[92]
		in vitro	MM55.K tubular cells	PTECs	8 µg/mL	48 h			-		
miR-685	Mouse	in vivo	Female Diversity Outbred mice	Urine and kidney	5 mg/kg	3 d	Toxic	Up	-	-	[26]
miR-702	Rat	in vivo	Male Sprague-Dawley rats	Plasma	2 or 5 mg/kg	72 h	Toxic	Up	-	-	[112]
miR-709	Mouse	in vivo	C57BL/6 mice	Blood and kidney	20 mg/kg	72 h	Toxic	Up	-	TFAM	[23]
		in vitro	mPTC cell line	PTECs	1–20 µM	2–24 h			-		
miR-741	Mouse	in vivo	Adult C57BL/6 mice	Urine, blood and kidney	15 mg/kg	4 d	Toxic	Up	-	-	[92]
		in vitro	MM55.K tubular cells	PTECs	8 µg/mL	48 h			-		
miR-1190	Mouse	in vivo	Adult C57BL/6 mice	Urine, blood and kidney	15 mg/kg	4 d	Toxic	Down	-	-	[92]
		in vitro	MM55.K tubular cells	PTECs	8 µg/mL	48 h			-		
miR-1839	Rat	in vivo	Male Sprague-Dawley rats	Urine	2 or 5 mg/kg	72 h	Toxic	Up	-	-	[112]
miR-6215	Rat	in vivo	Male Sprague-Dawley rats	Plasma	2 or 5 mg/kg	72 h	Toxic	Up	-	-	[112]
miR-let-7b	Rat	in vivo	Male Wistar rats	Blood and kidney	5 mg/kg	3 d	Toxic	Down	-	TGFβR-1, TAK1, mTOR, LC3-II	[90]
miR-let-7e	Rat	in vivo	Male Sprague-Dawley rats	Plasma	2 or 5 mg/kg	72 h	Toxic	Up	-	-	[112]
miR-let-7g	Rat	in vivo	Male Sprague-Dawley rats	Urine	2 or 5 mg/kg	72 h	Toxic	Up	-	-	[112]
miR-let-7g-5p	Rat	in vivo	Male Sprague-Dawley rats	Kidney and urine	1, 3 or 6 mg/kg	1–7 d	Toxic	Up	-	-	[114]

PTECs: Proximal Tubular Epithelial Cells.

## Data Availability

No new data were created or analyzed in this study. Data sharing is not applicable to this article.
